# Lysophosphatidic acid production and action: critical new players in breast cancer initiation and progression

**DOI:** 10.1038/sj.bjc.6605588

**Published:** 2010-03-16

**Authors:** N Panupinthu, H Y Lee, G B Mills

**Affiliations:** 1 Department of Systems Biology, The University of Texas M. D. Anderson Cancer Center, Houston, TX 77030, USA; 2 Department of Pharmacology, College of Medicine, Konyang University, Daejeon 302-718, Republic of Korea

**Keywords:** autotaxin, breast cancer, G protein-coupled receptor, inflammation, lysophosphatidic acid

## Abstract

Lysophosphatidic acid (LPA) is a potent lipid mediator that acts on a series of specific G protein-coupled receptors, leading to diverse biological actions. Lysophosphatidic acid induces cell proliferation, survival and migration, which are critically required for tumour formation and metastasis. This bioactive lipid is produced by the ectoenzyme lysophospholipase D or autotaxin (ATX), earlier known as an autocrine motility factor. The ATX–LPA signalling axis has emerged as an important player in many types of cancer. Indeed, aberrant expression of ATX and LPA receptors occurs during the development and progression of breast cancer. Importantly, expression of either ATX or LPA receptors in the mammary gland of transgenic mice is sufficient to induce the development of a high frequency of invasive and metastatic mammary cancers. The focus of research now turns to understanding the mechanisms by which ATX and LPA promote mammary tumourigenesis and metastasis. Targeting the ATX–LPA signalling axis for drug development may further improve outcomes in patients with breast cancer.

Breast cancer is heterogeneous in the development and progression with patients that have histologically indistinguishable tumours showing disparate outcomes. Breast cancer is the ‘poster child’ for personalised therapy with assessment of biomarkers, including oestrogen receptor (ER), progesterone receptor and human epidermal growth factor receptor 2 directing patients to specific targeted therapies. Although it is impossible to fully ascertain the relative function of earlier detection and improved therapy, mortality rates for breast cancer have shown remarkable improvement. Despite the marked improvements in outcomes, 192 370 new cases of breast cancer are predicted last year in the United States, with an expected death rate of ∼20% (Jemal *et al*, 2009). In terms of poor outcomes, therapy resistance and metastasis both remain as critical challenges. Therefore, additional therapeutic targets are required to improve outcomes. As the autotaxin (ATX)–lysophosphatidic acid (LPA) signalling axis is one of the important survival factors and contributes to invasion and metastasis (Mills and Moolenaar, 2003), it warrants investigation both in terms of its function in the initiation and progression of breast cancer and as a novel therapeutic target.

## The ATX–LPA signalling axis in cancer

Membrane lipids are critical for maintaining cell integrity and supporting multiple cell functions. The lipids themselves can also function directly as signalling molecules triggering profound cellular responses through high-affinity and selective cell-surface receptors. Lysophosphatidic acid (1- or 2-acyl-*sn*-glycero-3-phosphate), one of the simplest phospholipids, is produced and released by many different cell lineages including cancer cells. Despite its simple structure consisting of a glycerol backbone – a single fatty acyl chain and a single phosphate – there are many forms of LPA because of the structure, linkage and location of the fatty acyl chain on the glycerol backbone. These different forms of LPA showed distinct activities at different LPA receptors. In the extracellular fluid, LPA is derived from at least two mechanisms (Mills and Moolenaar, 2003). First, LPA can be produced by secretory phospholipase A_1_ or A_2_, which hydrolyses the fatty acid chain of phosphatidic acid at the *sn*-1 or *sn*-2 position, respectively. The other mechanism involves the hydrolysis of lysophosphatidylcholine by the lysophospholipase D activity of the ectoenzyme, ATX. Importantly, mice heterozygous for ATX have plasma LPA levels half that of normal mice (Tanaka *et al*, 2006; van Meeteren *et al*, 2006), suggesting that ATX represents the biologically relevant pathway determining plasma LPA levels.

The function of ATX in cancer progression was well documented before the demonstration that it exhibited lysophospholipase D activity. Autotaxin was identified as autocrine motility factor based on its function in stimulating chemokinesis and chemotaxis in melanoma cells (Stracke *et al*, 1992). Autotaxin was also shown to increase tumour invasiveness (Nam *et al*, 2000). Concurrently, many studies indicated LPA as an important mediator in cancer progression. Therefore, the discovery that ATX was the important enzyme producing LPA (Tokumura *et al*, 2002; Umezu-Goto *et al*, 2002) linked two independent research fields and catalysed a rapid advance in our knowledge of the function of the ATX–LPA signalling axis in cancer. Although ATX is a member of the nucleotide pyrophosphatase/phosphodiesterase family and had been proposed to mediate its activity as a nucleotide phosphodiesterase, it is now established that ATX mediates its functions primarily through production and action of LPA.

Lysophosphatidic acid binds to a series of high-affinity cell-surface G protein-coupled receptors (GPCRs). There are at least six GPCRs currently identified as LPA receptors, designated LPA1-6 (Noguchi *et al*, 2009; Yanagida *et al*, 2009). Other putative GPCRs that seem to bind and respond to LPA also have been identified (Tabata *et al*, 2007; Murakami *et al*, 2008); however, further characterisation as *bona fide* LPA receptors is still needed. The best-characterised LPA receptors belong to the endothelial differentiation gene (EDG) family – LPA1/EDG2, LPA2/EDG4 and LPA3/EDG7 receptors that also include a series of receptors for the related sphingosine 1-phosphate. Interestingly, at least one putative LPA receptor is proposed to respond to both LPA and sphingosine 1-phosphate (Murakami *et al*, 2008). Recently, these EDG-family LPA receptors as well as ATX have been implicated in the development and progression of breast cancer (Liu *et al*, 2009).

## Aberrant expression of ATX or the EDG-family LPA receptors is sufficient to drive mammary tumourigenesis

Expression of ATX and the EDG-family LPA receptors is upregulated in many types of cancer. Autotaxin is highly expressed and correlated with outcomes in a number of cancer lineages, including glioblastoma (Kishi *et al*, 2006) and prostate cancer (Nouh *et al*, 2009). Breast cancer cells express ATX transcripts at a higher level compared with normal breast epithelium (Yang *et al*, 2002). Interestingly, aberrant expression of ATX markedly enhanced the aggressiveness of breast cancer cells (Yang *et al*, 2002).

Transformation of normal cells may be mediated, in part, by the upregulation of ATX and the EDG-family LPA receptors. For example, ATX expression was upregulated ∼100-fold in viral Jun oncogene-transformed chick embryonic fibroblasts (Black *et al*, 2004). In contrast, the tumour suppressor Nm23-H1 downregulated expression of LPA1 in MDA-MB-435 cells (Horak *et al*, 2007). Although the functions of Jun and Nm23-H1 have been implicated in breast cancer, whether their effects are mediated through the ATX–LPA axis remains to be ascertained.

The EDG family of LPA receptors are expressed by normal mammary epithelial cells with aberrant expression of the EDG-family LPA receptors occurring during breast cancer initiation and progression. The function of LPA1 in the progression of breast cancer has been studied more extensively than that of other LPA receptors. Over-expression of LPA1 is readily observed in breast cancer cells, suggesting a function in transformation (Witt *et al*, 2006). Further, a number of breast cancer cell lines express high levels of LPA1 transcripts (Chen *et al*, 2007). Manipulation of LPA1 levels or function in breast cancer cell lines altered the ability of breast cancer cell lines to metastasise to bone (Boucharaba *et al*, 2004, 2006). We have recently shown that expression of LPA1 in mammary epithelial cells of transgenic mice was sufficient to induce the development of mammary cancers with a significant proportion being invasive and metastatic (Liu *et al*, 2009). However, the late onset of tumour development suggests that additional events must occur for the manifestation of the effects of transgenic expression of LPA1. Whether these are stochastic events or are driven by the expression of the LPA1 receptor in a cell-autonomous manner will require additional exploration. Nevertheless, the combined data validates LPA1 as a high-quality therapeutic target for drug development and evaluation in breast cancer.

Less is known about the functions of LPA2 and LPA3 in breast cancer pathophysiology. Similar to ATX and LPA1, increased LPA2 expression has been associated with tumour invasiveness (Kitayama *et al*, 2004) and breast cancer progression (Li *et al*, 2009). Transgenic mice over-expressing LPA2 developed mammary tumours with the onset and frequency similar to those observed in LPA1 transgenic mice (Liu *et al*, 2009), further implicating LPA2 in the initiation and progression of breast cancer. In the central nervous system, LPA1 and LPA2 seem to mediate redundant functions during brain development (Contos *et al*, 2002). The similar phenotypes of transgenic LPA1 and LPA2 mice suggest that these receptors may also mediate similar functions during breast cancer initiation and progression. Interestingly, LPA2-deficient mice are resistant to the induction of colon cancer (Lin *et al*, 2009). Whether LPA2 (or LPA1)-deficient mice are resistant to the development of breast cancer remains to be determined.

It is not clear whether expression of LPA3 is altered during breast cancer progression. Lysophosphatidic acid-3 is less abundant than LPA1 or LPA2 in breast cancer cells (Chen *et al*, 2007) and did not seem to be altered in patients with metastatic breast cancer (Kitayama *et al*, 2004). However, publicly available microarray data sets show that poorly differentiated breast cancers showed higher expression of LPA2 and LPA3 than well-differentiated tumours (Liu *et al*, 2009). As poorly differentiated breast cancers have a worsened outcome, it is possible that LPA3 contributes to breast cancer pathophysiology. In support of this contention, transgenic expression of LPA3 driven by the mouse mammary tumour virus promoter resulted in tumour development and metastasis similar to that observed in LPA1 or LPA2 transgenic mice (Liu *et al*, 2009). Together, the data from transgenic mouse models indicated that each of the EDG family of LPA receptors is sufficient to induce invasive and metastatic tumours when aberrantly expressed in mammary epithelial cells.

## Mechanisms underlying mammary tumourigenesis induced by ATX and the EDG-family LPA receptors

**Figure 1:**
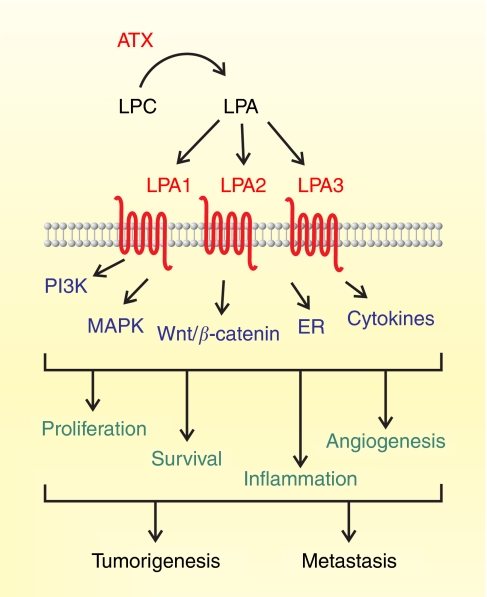
Current understanding of breast cancer progression mediated by ATX–LPA signalling axis. Autotaxin and LPA1–3 receptors are expressed in mammary glands and can exert multiple effects under physiological conditions. Lysophosphatidic acid is produce from lysophosphatidylcholine (LPC) by ATX and acts on the EDG-family LPA receptors, LPA1, LPA2 and LPA3. As an important pathway promoting cell survival, the ATX–LPA signalling axis may initiate tumourigenesis in breast by allow cells to be susceptible to other genetic mutations, leading to accumulation of several aberrant signalling pathways. Indeed, each of these components of the ATX–LPA signalling axis sufficiently induces tumourigenesis through upregulation of many signalling pathways, including PI3K, MAPK, Wnt/*β*-catenin and ER. In addition, marked increase in the production of several cytokines by LPA further advance the disease progression by local inflammation and angiogenesis. The effects of LPA on cytokine production and blood vessel formation may contribute to the metastasis of breast tumours to other tissues such as bone.

The mechanisms underlying LPA-driven tumour development in mammary glands remains to be fully elucidated despite extensive evidence implicating LPA in processes and pathways associated with tumourigenesis *in vitro* (Figure 1). Interestingly, tumour development induced by ATX and the EDG-family LPA receptors commonly displayed late onset ranging from 8 to 24 months and occurred in a subpopulation of each transgenic line (Liu *et al*, 2009). The late onset and stochastic tumour development suggest that expression of ATX or the EDG-family LPA receptors cooperates with other events such as secondary mutations to generate the full tumourigenic phenotype. Indeed, when these tumours were profiled at both the transcript and protein levels, they did not form a clear cluster similar to the tight clusters observed in tumours driven by a number of known oncogenes. This suggests that multiple different events cooperate with expression of ATX or the EDG family of receptors in inducing tumourigenesis. Nevertheless, the tumours did show the activation of signalling pathways that have been associated with signalling through LPA receptors *in vitro*, thus suggesting that LPA signalling is functional and likely contributes to both the initiation and progression of the tumours.

A number of important questions require further attention. In particular, it will be critical to determine whether the effects of ATX and the EDG-family LPA receptors are cell autonomous solely through activating process in the transgenic epithelial cells. Alternatively, ATX or cytokines produced by the transgenic epithelial cells could alter functions of the stroma, which in turn contributes to the tumourigenic process. Indeed, in many cases, tumour development in the ATX and LPA receptor transgenic mice was preceded by chronic inflammation of the mammary glands and by elevated levels of circulating cytokines (Liu *et al*, 2009), compatible with a bidirectional interaction between the epithelium and stromal cells contributing to tumourigenesis. If this question is fully addressed, the outcome could suggest a number of different interventions that would potentially decrease breast cancer initiation and progression.

It is striking that expression of ATX or each of the EDG-family LPA receptors resulted in an increased incidence of invasive and metastatic mammary tumours albeit at different frequencies and onsets. This suggests that each of the LPA receptor subtypes mediates common processes sufficient to support the initiation and progression of tumours. A similar ability to alter tumourigenic behaviour was also found when expression of the LPA receptors was elevated in human cancer cell lines. This showed a marked overlap in the ability of LPA1, LPA2 or LPA3 to initiate tumourigenesis despite earlier evidence that each of the LPA receptors coupled to different signalling pathways (Yu *et al*, 2008).

Optimal activation of LPA receptors requires ligation by LPA. Thus, it is likely that the initial step of tumourigenesis induced by over-expression of the LPA receptors involves endogenous LPA produced locally by ATX acting on transgenic LPA receptors on the breast epithelium. Nevertheless, it is possible that the transgenic LPA receptors are present at sufficiently high levels to result in a ligand-independent activation through the formation of homo- or heterodimers in the absence of exogenous LPA. In terms of the function of the ATX transgene in tumour progression, it is also likely that the elevated level of LPA activates endogenous LPA receptors present in the epithelium or surrounding stroma. In many tissues including breast, LPA levels are regulated through the balance of its local production by ATX and degradation by lipid phosphatases. Indeed, transgenic expression of ATX under a liver-specific promoter was not sufficient to induce mammary or other tumours despite elevated circulating LPA levels (Pamuklar *et al*, 2009).

Although ATX and the EDG-family LPA receptors are expressed in mammary epithelial and stromal cells of both mouse (Liu *et al*, 2009) and human (Finak *et al*, 2008), and expression levels are altered during breast tumourigenesis, little attention has been given to the mechanisms underlying the tumorigenesis by the ATX-LPA axis. The stress associated with acquisition of oncogenic events or loss of tumour suppressors can result in cell death or the induction of senescence, a process designated ‘oncogenic stress’. However, the ability of LPA to enhance cell survival may allow secondary mutations to be ‘fixed’ in the genome and for cells to continue to proliferation despite ‘oncogenic stress’. In this regard, it has been shown that activation of LPA2 suppressed p53-mediated senescence (Kortlever *et al*, 2008). Lysophosphatidic acid-2 as well as LPA1 and LPA4 receptors also act cooperatively with c-Myc oncogene or with the immortalising gene TBX2 to inhibit apoptosis and induce transformation of embryonic fibroblasts (Taghavi *et al*, 2008). Amplification of TBX2 has been reported in a subset of breast cancer patients (Jacobs *et al*, 2000); however, a direct link between LPA signalling and TBX2 levels has not been shown in breast cancer. Together, it is conceivable that signalling pathways activated by the ATX–LPA axis initiate tumour development in breast, in part, by extending cell survival and allowing cells to be susceptible to the effects of genetic mutations affecting other genes. If this is the case, the ATX and LPA receptor transgenic mice may recapitulate the heterogeneity of tumours found in patients with breast cancer. Indeed, marked heterogeneity of proteomic and transcriptional profiles was observed in the tumours from the transgenic mice.

## Signalling pathways associated with the ATX–LPA signalling axis in breast cancer

Lysophosphatidic acid elicits many biological effects, including cell proliferation, survival and migration, that is indeed essential for the progression of cancer (Mills and Moolenaar, 2003). Lysophosphatidic acid binds to LPA receptors coupled with at least three subtypes of G proteins, G_q_, G_i_ and G_12/13_, leading to the activation of multiple downstream signalling pathways (Figure 1). Mammary tumours derived from transgenic mice expressing ATX or each of the EDG-family LPA receptors showed upregulation and activation of downstream pathways associated with receptor tyrosine kinase signalling, including the PI3K/Akt, p38-MAPK and ERK/MAPK pathways (Liu *et al*, 2009). Aberrant receptor tyrosine kinase function is an important hallmark of breast cancer. As LPA activates a number of tyrosine kinase receptors by increasing the rates of release of the cell-surface-tethered growth factors (Mills and Moolenaar, 2003), it is possible that LPA receptors promote breast cancer progression through the transactivation of cell-surface tyrosine kinase receptors.

Activation of the Ras/Raf/MAPK pathway has been linked to the progression of breast cancer (Caldas-Lopes *et al*, 2009). In addition, the presence of cyclin D1 was essential for Ras-induced transformation of mammary epithelial cells (Yu *et al*, 2001). Interestingly, upregulation of MAPK and cyclin D1 was found in mammary tumours induced by ATX or the EDG-family LPA receptors (Liu *et al*, 2009). Therefore, LPA may activate the Ras/MAPK/cyclin D1 pathway to modulate cell-cycle progression and contribute to the transformation of mammary epithelial cells. Recent evidence suggests that LPA promotes motility and invasiveness of breast cancer cells though the Ral GTPase, a member of the Ras superfamily (Li *et al*, 2009). It will thus be important to determine the relative functions of the Ral GTPase and the Ras/MAPK/cyclin D1 pathway in the invasion and metastasis observed in the ATX and LPA receptor transgenic mice.

The presence of ER is critical for the development and progression of the majority of breast cancers in human. However, tumours from most mouse models of breast cancer are ER negative. Interestingly, a subset of tumours in the transgenic mouse models of ATX and LPA receptors expressed ER and, in particular, phosphorylated ER in the nucleus (Liu *et al*, 2009), suggesting that ER has a function in the development of LPA-induced breast cancer at least in the ER-positive tumours. However, the mechanisms linking ATX and LPA to ER expression remain unknown. The variable ER expression could relate to the transformation of a different precursor cell or to differential regulation of ER expression. The presence of the activated ER in a subset of the transgenic tumours suggests that combination therapy targeting ER and ATX–LPA signalling may be effective for treatment and improve prognosis in patients with ER-positive breast cancer.

The canonical Wnt/*β*-catenin signalling is critical for the maintenance of epithelial progenitor cell populations (Reya and Clevers, 2005). On the other hand, disruption of this signalling cascade leads to epithelial transformation and tumourigenesis. Over-expression of N-terminal-truncated *β*-catenin in mammary epithelial cells leads to the development of basal-type carcinoma in the multiparous mice (Teuliere *et al*, 2005). Interestingly, it has been shown that LPA-induced proliferation of HCT116 and LS174T colon cancer cells require phosphorylation and nuclear translocation of *β*-catenin through phosphorylation and inhibition of GSK-3*β* (Yang *et al*, 2005). Activation of Wnt signalling by LPA has also been shown in breast cancer cells by suppression of GSK-3*β* and accumulation of *β*-catenin in the nucleus (Liu *et al*, 2009). Therefore, development of breast tumours driven by the ATX–LPA signalling may require activation of the canonical Wnt/*β*-catenin pathway to induce the transformation of mammary epithelial cells. Conversely, it is possible that the Wnt/*β*-catenin signalling regulates the ATX–LPA signalling axis and contributes to the progression of breast cancer (Malbon, 2005).

## breast cancer progression may involve LPA-induced cytokine production

Chronic inflammation is a common observation during cancer progression and is associated with marked production of cytokines that could further aggravate tumour progression. Indeed, the ATX–LPA signalling axis has been linked to cytokine production and chronic inflammation through autocrine or paracrine mechanisms (Yu *et al*, 2008; Liu *et al*, 2009). Elevated LPA production induced by ATX has also been observed in chronic hepatitis (Watanabe *et al*, 2007). Interestingly, LPA2 receptors were required for tumour initiation and progression in an inflammation-induced mouse model of colon cancer (Lin *et al*, 2009). Together, this suggests that the production and actions of LPA have crucial functions in both inflammation and tumour initiation.

Systemic inflammation has been associated with an increased risk of breast cancer development (Gadalla *et al*, 2009) and metastasis (Das Roy *et al*, 2009). In addition, local inflammation leading to chronic mastitis in the mammary gland occurred at a higher frequency and earlier time point of tumour development in the ATX and LPA receptor transgenic mice (Liu *et al*, 2009). Inflammatory cytokines such as interleukin 6 and interleukin 8 have been implicated in bone metastasis (Boucharaba *et al*, 2004). In accord, these cytokines were elevated before the development of breast tumours and markedly increased in a subset of tumour-bearing mice (Liu *et al*, 2009). Thus, production of inflammatory cytokines as a consequence of expression of ATX and LPA receptors in the transgenic mice may contribute to tumourigenesis by inducing sustained inflammation of mammary glands (Figure 1). Whether the induction of inflammation and tumourigenesis is orthologous events or whether the ATX–LPA signalling axis in epithelial cells results in the activation of inflammatory cells that contribute to tumourigenesis will need to be analysed through experimental interventions. Further, it will be critically important to determine whether the activation of ATX and LPA receptors induces production of inflammatory cytokines in a cell-autonomous manner or through bidirectional interaction between the epithelial cells and the microenvironment.

The ‘angiogenic switch’ has been proposed to represent an important transition from the benign to the malignant state. The ATX and LPA stimulate production of many angiogenic factors including vascular endothelial growth factor in ovarian cancer cells (Hu *et al*, 2001; Lee *et al*, 2006; Ptaszynska *et al*, 2008). In breast tumours driven by ATX and LPA receptors, levels of vascular endothelial growth factor were also increased before the presence of detectable tumours with levels increasing in mice with extant tumours (Liu *et al*, 2009). Thus, the ATX–LPA axis may contribute to the angiogenic switch in breast cancer that leads to tumour invasion and metastasis.

## Future prospects for the ATX–LPA signalling axis as a target in breast cancer

As we first implicated LPA as a potential mediator in breast tumourigenesis by studies of breast cancer cell lines (Xu *et al*, 1995), many subsequent studies have combined to establish an important function for the ATX–LPA axis in the development and progression of breast cancer. The demonstration that expression of ATX and the EDG-family LPA receptors is sufficient to induce mammary tumours suggests that the ATX–LPA signalling axis is a novel target in breast cancer and potentially other cancer lineages. Multiple approaches targeting specific components of the ATX–LPA signalling axis are in preclinical development. Silencing LPA1 expression by siRNA or inhibiting LPA function with small molecule inhibitors effectively suppressed cytokine production and bone metastasis of breast cancer cell lines (Boucharaba *et al*, 2006). Interestingly, LPA analogues, which have dual activities as ATX and LPA receptor inhibitors suppressed migration of breast cancer cells *in vitro* as well as tumour growth *in vivo* (Zhang *et al*, 2009). In other systems, selective ATX inhibitors reduced metastasis of melanoma cells to lung (Baker *et al*, 2006). These and other ATX inhibitors should be tested in breast cancer models. Indeed, these studies are underway in the transgenic mouse models. In addition, a monoclonal antibody that specifically binds and neutralises LPA has been developed and is in late preclinical development (http://www.lpath.com). Together, these approaches should be evaluated as potential additions to the armamentarium for the treatment of breast cancer.
